# Supplementary material for: Pharmaceutical cost dynamics for the treatment of rifampicin-resistant tuberculosis in children and adolescents in South Africa, India, and the Philippines
Source: PLoS One. 2024 Jul 23;19(7):e0305930. doi: 10.1371/journal.pone.0305930 (PMC11265651; doi:10.1371/journal.pone.0305930)
Supplement: S1 File — (DOCX) [file pone.0305930.s001.docx]

**Supplementary appendix:**

Pharmaceutical cost dynamics for the treatment for rifampicin-resistant tuberculosis in children and adolescents in South Africa, India, and the Philippines

Contents

[APPENDIX 1: [TABLE] UNIT COST OF MEDICINES INCLUDED IN ANALYSIS BY FORMULATION, PRICE SOURCE AND DOSING 2](#_Toc148700173)

[APPENDIX 2A: [TABLE] COST PER MONTH OF RR-TB MEDICINES USING CHILD-FRIENDLY FORMULATIONS BY AGE AND WEIGHT BAND 4](#_Toc148700174)

[APPENDIX 2B: [TABLE] COST PER MONTH OF RR-TB MEDICINES USING ADULT FORMULATIONS BY AGE AND WEIGHT BAND 5](#_Toc148700175)

[APPENDIX 3: [TABLE] AVERAGE MONTHLY COST (US$) FOR RR-TB PAEDIATRIC REGIMENS BY SETTING, FORMULATION AND AGE 6](#_Toc148700176)

[APPENDIX 4: [TABLE] TOTAL COST BY NATIONAL GUIDANCE FOR RR-TB PEDIATRIC REGIMENS, PPP-ADJUSTED 8](#_Toc148700177)

[APPENDIX 5: [TABLE] TOTAL COST OF WHO RECOMMENDED RR-TB PAEDIATRIC REGIMEN IN SOUTH AFRICA, INDIA AND PHILIPPINES*,* PPP ADJUSTED 10](#_Toc148700178)

[APPENDIX 6: [FIGURE] PROPORTIONAL CONTRIBUTION OF INDIVIDUAL CHILD-FRIENDLY AND ADULT FORMULATIONS TO TOTAL PAEDIATRIC RR-TB REGIMEN COST 11](#_Toc148700179)

[APENDIX 7: [FIGURE] WHO RR-TB REGIMEN COST ($I) AS A FACTOR OF DOMESTIC GENERAL GOVERNMENT HEALTH EXPENDITURE, PER CAPITA (GDF PROCUREMENT, PPP ADJUSTED in <15 YEARS AGE GROUP) 12](#_Toc148700180)

[APPENDIX 8: SOURCE GUIDANCE FOR INCLUDED REGIMEN: 13](#_Toc148700181)

[APPENDIX 9: [TABLE] PROPORTION OF POPULATION FALLING IN WEIGHT BANDS USED IN THE ANALYSIS: 17](#_Toc148700182)

# APPENDIX 1: [TABLE] UNIT COST OF MEDICINES INCLUDED IN ANALYSIS BY FORMULATION, PRICE SOURCE AND DOSING

| **Medicine and formulation** | **GDF: cost per unit (US$)** | **MHPL: cost per unit (US$)** | **Wastage assumption** | **Included in analysis** | | **Reason if not included in analysis** |
| --- | --- | --- | --- | --- | --- | --- |
|  |  |  |  | **Child formulation** | **Adult formulation** |  |
| BDQ 20mg tablet | 0.43 | Not listed | Tablet can be halved | x |  |  |
| BDQ 100mg tablet | 0.65 | 1.02 | Tablet cannot be halved |  | x |  |
| CFZ 50mg tablet | 0.40 | Not listed | Not relevant |  |  | x - 50mg capsule available at lower cost |
| CFZ 50mg capsule | 0.36 | Not listed | Capsule cannot be halved | x |  |  |
| CFZ 100mg capsule or tablet | 0.65 | 0.58 | Capsules or tablet cannot be halved |  | x |  |
| CS 125mg capsule | 0.45 | Not listed | Capsule cannot be halved | x |  |  |
| CS 250mg capsule | 0.25 | Not listed | Capsule cannot be halved |  | x |  |
| DLM 25mg dispersible tablet | 1.77 | Not listed | Dispersible tablet can be halved | x |  |  |
| DLM 50mg tablet | 1.77 | 2.04 | Tablet cannot be halved |  | x |  |
| E 100mg dispersible tablet | 0.21 | Not listed | Not relevant |  |  | x - 100mg tablet available at lower cost |
| E 100mg tablet | 0.04 | Not listed | Tablet can be halved | x |  |  |
| E 400mg tablet | 0.04 | 0.05 | Tablet can be halved |  | x |  |
| ETO 125mg dispersible tablet | 0.14 | Not listed | Dispersible tablet can be halved | x |  |  |
| ETO 250mg tablet | 0.09 | 0.16 | Tablet cannot be halved |  | x |  |
| H 100mg dispersible tablet | 0.09 | Not listed | Not relevant |  |  | X - 100mg tablet available at lower cost |
| H 100mg tablet | 0.01 | 0.03 | Tablet can be halved | x |  |  |
| H 300mg tablet | 0.02 | 0.04 | Tablet can be halved |  | x |  |
| LFX 100mg dispersible tablet | 0.12 | Not listed | Dispersible tablet can be halved | x |  |  |
| LFX 250mg tablet | 0.03 | 0.13 | Tablet can be halved |  | x |  |
| LZD 20mg/ml oral suspension | Not listed | 0.84 | Not relevant |  |  | x - not available on GDF |
| LZD 150mg dispersible tablet | 0.27 | Not listed | Dispersible tablet can be halved | x |  |  |
| LZD 600mg tablet | 0.19 | 0.58 | Tablet can be halved |  | x |  |
| MFX 100mg dispersible tablet | 0.20 | Not listed | Dispersible tablet can be halved | x |  |  |
| MFX 400mg tablet | 0.16 | 0.25 | Tablet cannot be halved |  | x |  |
| PAS sodium salt 4g powder sachet | 1.32 | 2.3 | Calculated number of sachets needed for total daily dose | x | x |  |
| PA 200mg tablet | 1.31 | Not listed | Tablet cannot be halved | x | x |  |
| PTO 250mg tablet | 0.09 | Not listed |  | x~ | x |  |
| TRD 250mg capsule | 1.75 | 0.98 | Capsule cannot be halved | x~ | x |  |
| Z 150mg dispersible tablet | 0.15 | Not listed | Dispersible tablet can be halved | x |  |  |
| Z 400mg tablet | 0.021 | Not listed | Not relevant |  |  | x - 500mg tablet available at lower cost |
| Z 500mg tablet | 0.020 | 0.06 | Tablet can be halved |  | x |  |
| Pyr(B6) 10mg tablet | 0.03 | Not listed | Not relevant |  |  | x - 50mg tablet available at lower cost on GDF |
| Pyr(B6) 25mg tablet | Not listed | 0.007 | Tablet can be halved | x (MHPL analysis) | x (MHPL analysis) |  |
| Pyr(B6) 50mg tablet | 0.01 | Not listed | Tablet can be halved | x (GDF analysis) | x (GDF analysis) |  |

BDQ-bedaquiline, CFZ-clofazimine, CS-cycloserine, DLM-delamanid, E-ethambutol, ETO-ethionamide, hH-high dose isoniazid, LFX-levofloxacin, LZD-linezolid, MFX-moxifloxacin, , PAS-para-aminosalicylic acid, PA-Pretomanid, PTO-prothionamide, Pyr(B6)-Pyridoxine, TRD-terizidone, Z-pyrazinamide

GDF-Global Drug Facility (October 2023); MHPL-Master Health Product List (South African National Tender pricing, February 2024). In some instances the February 2024 MHPL has not updated prices for some formulations that are currently supplied, in which case the August 2023 version of the MHPL was used.

US$ = United States dollar (not adjusted for purchasing power parity)

~There are no child-friendly formulations of PTO and TRD available, so the adult dose was used in the analysis of child-friendly preparations when applicable.

# APPENDIX 2A: [TABLE] COST PER MONTH OF RR-TB MEDICINES USING CHILD-FRIENDLY FORMULATIONS BY AGE AND WEIGHT BAND

| **Drugs and formulations** | **Cost (US$) per month in children aged:** | | **Cost (US$) per month by weight group (kg)** | | | | | | | | | | |
| --- | --- | --- | --- | --- | --- | --- | --- | --- | --- | --- | --- | --- | --- |
|  | **<15 years old** | **<5 years old** | **3 to <5** | **5 to <7** | **7 to <10** | **10 to <12** | **12 to <16** | **16 to <20** | **20 to <24** | **24 to <30** | **30 to <36** | **36 to <46** | **46 and over** |
| **Child-friendly formulations used in the analysis** | | | | | | | | | | | | | |
| BDQ 20mg_month1 | 83.00 | 39.05 | 10.21 | 15.32 | 28.93 | 43.40 | 43.40 | 72.34 | 72.34 | 72.34 | 144.67 | 144.67 | 144.67 |
| BDQ 20mg_month2 onwards | 29.24 | 13.64 | 2.55 | 3.83 | 10.21 | 15.32 | 15.32 | 25.53 | 25.53 | 25.53 | 51.06 | 51.06 | 51.06 |
| BDQ 20mg_month1 (potential future dosing) | 102.49 | 48.14 | 11.91 | 17.87 | 35.74 | 53.61 | 53.61 | 89.36 | 89.36 | 89.36 | 178.71 | 178.71 | 178.71 |
| BDQ 20mg_month2 onwards  (potential future dosing) | 68.24 | 31.82 | 5.96 | 8.94 | 23.83 | 35.74 | 35.74 | 59.57 | 59.57 | 59.57 | 119.14 | 119.14 | 119.14 |
| LFX 100mg dt | 14.76 | 6.24 | 1.70 | 3.40 | 5.10 | 6.80 | 6.80 | 10.19 | 10.19 | 16.99 | 25.48 | 25.48 | 33.98 |
| MFX 100mg dt | 16.72 | 10.15 | 2.77 | 5.53 | 8.30 | 11.06 | 11.06 | 16.59 | 16.59 | 22.13 | 22.13 | 22.13 | 19.76 |
| LZD 150mg dt | 14.58 | 7.74 | 3.82 | 3.82 | 7.64 | 7.64 | 7.64 | 15.27 | 15.27 | 15.27 | 15.27 | 22.91 | 30.54 |
| CFZ 50mg cap | 13.74 | 8.12 | 2.86 | 4.29 | 4.29 | 10.02 | 10.02 | 10.02 | 10.02 | 20.04 | 20.04 | 20.04 | 20.04 |
| CS 125mg cap | 39.04 | 21.96 | 12.60 | 12.60 | 12.60 | 25.20 | 25.20 | 37.80 | 37.80 | 50.40 | 50.40 | 50.40 | 75.60 |
| TRD 250mg cap | 84.69 | 52.22 | 49.00 | 49.00 | 49.00 | 49.00 | 49.00 | 98.00 | 98.00 | 98.00 | 98.00 | 98.00 | 147.00 |
| E 100mg tab | 5.20 | 2.63 | 0.50 | 1.00 | 2.00 | 3.01 | 3.01 | 4.01 | 4.01 | 6.01 | 8.02 | 8.02 | 12.03 |
| DLM 25mg dt | 155.69 | 99.01 | 49.58 | 74.38 | 99.17 | 99.17 | 99.17 | 148.75 | 148.75 | 148.75 | 198.33 | 198.33 | 396.67 |
| Z 150mg dt | 24.10 | 11.45 | 2.13 | 4.26 | 8.52 | 12.78 | 12.78 | 21.31 | 21.31 | 25.57 | 34.09 | 42.62 | 42.62 |
| ETO 125mg dt | 12.05 | 6.78 | 3.89 | 3.89 | 3.89 | 7.78 | 7.78 | 11.67 | 11.67 | 15.56 | 15.56 | 15.56 | 23.34 |
| PTO 250mg tab | 4.45 | 2.75 | 2.58 | 2.58 | 2.58 | 2.58 | 2.58 | 5.15 | 5.15 | 5.15 | 5.15 | 5.15 | 7.73 |
| PAS 4g sachet | 62.26 | 39.39 | 36.96 | 36.96 | 36.96 | 36.96 | 36.96 | 73.92 | 73.92 | 73.92 | 73.92 | 73.92 | 73.92 |
| PA 200mg tab | 36.56 | 36.56 | 36.56 | 36.56 | 36.56 | 36.56 | 36.56 | 36.56 | 36.56 | 36.56 | 36.56 | 36.56 | 36.56 |
| hH 100mg tab | 1.13 | 0.64 | 0.18 | 0.35 | 0.53 | 0.70 | 0.70 | 1.05 | 1.05 | 1.41 | 1.41 | 1.58 | 2.11 |
| Pyr (B6) 50mg tab | 0.22 | 0.18 | 0.18 | 0.18 | 0.18 | 0.18 | 0.18 | 0.18 | 0.18 | 0.18 | 0.18 | 0.37 | 0.37 |

Pricing source: Global Drug Facility October 2023;

Costs represented reflect mean average within stated age or weight category.

Daily dosing recommendations: WHO operational handbook on tuberculosis. Module 5: management of tuberculosis in children and adolescents, 2022

DQ-bedaquiline, CFZ-clofazimine, CS-cycloserine, DLM-delamanid, E-ethambutol, ETO-ethionamide, hH-high dose isoniazid, LFX-levofloxacin, LZD-linezolid, MFX-moxifloxacin, PA-pretomanid, PAS-para-aminosalicylic acid, PTO-prothionamide, Pyr(B6)-Pyridoxine, TRD-terizidone, Z-pyrazinamide Tab: tablet; cap: capsule; dt: dispersible tablet

WHO: World Health Organization; GDF: Global Drug Facility; US$: United States Dollar (not adjusted for purchasing power parity); RR-TB: Rifampicin- resistant tuberculosis

# APPENDIX 2B: [TABLE] COST PER MONTH OF RR-TB MEDICINES USING ADULT FORMULATIONS BY AGE AND WEIGHT BAND

| **Drugs and formulations** | **Cost (US$) per month in children aged:** | | **Cost (US$) per month by weight group (kg)** | | | | | | | | | | |
| --- | --- | --- | --- | --- | --- | --- | --- | --- | --- | --- | --- | --- | --- |
|  | **<15 years old** | **<5 years old** | **3 to <5** | **5 to <7** | **7 to <10** | **10 to <12** | **12 to <16** | **16 to <20** | **20 to <24** | **24 to <30** | **30 to <36** | **36 to <46** | **46 and over** |
| **Adult formulations used in the analysis** | | | | | | | | | | | | | |
| BDQ 100mg month 1 | 27.80 | 19.16 | 13.00 | 13.00 | 13.00 | 22.10 | 22.10 | 22.10 | 22.10 | 22.10 | 44.20 | 44.20 | 44.20 |
| BDQ 100mg month2 onwards | 10.16 | 7.80 | 7.80 | 7.80 | 7.80 | 7.80 | 7.80 | 7.80 | 7.80 | 7.80 | 15.60 | 15.60 | 15.60 |
| BDQ 20mg month1 (potential future dosing) | 34.58 | 24.36 | 18.20 | 18.20 | 18.20 | 27.30 | 27.30 | 27.30 | 27.30 | 27.30 | 54.60 | 54.60 | 54.60 |
| BDQ 20mg month2 onwards  (potential future dosing) | 23.71 | 18.20 | 18.20 | 18.20 | 18.20 | 18.20 | 18.20 | 18.20 | 18.20 | 18.20 | 36.40 | 36.40 | 36.40 |
| LFX 250mg tab | 1.45 | 0.68 | 0.39 | 0.39 | 0.39 | 0.78 | 0.78 | 1.18 | 1.18 | 1.57 | 2.35 | 2.35 | 3.14 |
| MFX 400mg tab | 4.34 | 4.34 | 4.34 | 4.34 | 4.34 | 4.34 | 4.34 | 4.34 | 4.34 | 4.34 | 4.34 | 4.34 | 4.34 |
| LZD 600mg tab | 3.22 | 2.69 | 2.69 | 2.69 | 2.69 | 2.69 | 2.69 | 2.69 | 2.69 | 2.69 | 2.69 | 5.38 | 5.38 |
| CFZ 100mg tab | 12.12 | 7.00 | 5.23 | 5.23 | 5.23 | 7.85 | 7.85 | 7.85 | 7.85 | 18.31 | 18.31 | 18.31 | 18.31 |
| CS 250mg cap | 12.20 | 7.52 | 7.06 | 7.06 | 7.06 | 7.06 | 7.06 | 14.11 | 14.11 | 14.11 | 14.11 | 14.11 | 21.17 |
| TRD 250mg cap | 84.69 | 52.22 | 49.00 | 49.00 | 49.00 | 49.00 | 49.00 | 98.00 | 98.00 | 98.00 | 98.00 | 98.00 | 147.00 |
| E 400mg tab | 1.41 | 0.60 | 0.56 | 0.56 | 0.56 | 0.56 | 0.56 | 1.12 | 1.12 | 1.68 | 2.25 | 2.25 | 3.37 |
| DLM 50mg tab | 102.37 | 95.75 | 49.58 | 74.38 | 99.17 | 99.17 | 99.17 | 99.17 | 99.17 | 99.17 | 99.17 | 99.17 | 198.33 |
| Z 500mg tab | 0.97 | 0.50 | 0.28 | 0.28 | 0.28 | 0.57 | 0.57 | 0.85 | 0.85 | 1.14 | 1.14 | 1.71 | 1.71 |
| ETO 250mg tab | 4.43 | 2.73 | 2.56 | 2.56 | 2.56 | 2.56 | 2.56 | 5.13 | 5.13 | 5.13 | 5.13 | 5.13 | 7.69 |
| PTO 250mg tab | 4.45 | 2.75 | 2.58 | 2.58 | 2.58 | 2.58 | 2.58 | 5.15 | 5.15 | 5.15 | 5.15 | 5.15 | 7.73 |
| PAS 4g sachet | 62.26 | 39.39 | 36.96 | 36.96 | 36.96 | 36.96 | 36.96 | 73.92 | 73.92 | 73.92 | 73.92 | 73.92 | 73.92 |
| PA 200mg tab | 36.56 | 36.56 | 36.56 | 36.56 | 36.56 | 36.56 | 36.56 | 36.56 | 36.56 | 36.56 | 36.56 | 36.56 | 36.56 |
| H 300mg tab | 0.61 | 0.43 | 0.26 | 0.26 | 0.26 | 0.51 | 0.51 | 0.51 | 0.51 | 0.77 | 0.77 | 0.77 | 1.03 |
| Pyr(B6) 50mg tab | 0.22 | 0.18 | 0.18 | 0.18 | 0.18 | 0.18 | 0.18 | 0.18 | 0.18 | 0.18 | 0.18 | 0.37 | 0.37 |
| **Additional formulations*** | | | | | | | | | | | | | |
| CFZ 50mg tab | 15.51 | 9.17 | 3.23 | 4.85 | 4.85 | 11.31 | 11.31 | 11.31 | 11.31 | 22.62 | 22.62 | 22.62 | 22.62 |
| E 100mg dt | 30.47 | 15.40 | 2.94 | 5.87 | 11.75 | 17.62 | 17.62 | 23.50 | 23.50 | 35.25 | 47.00 | 47.00 | 70.49 |
| Pyr(B6) 10mg tab | 1.79 | 0.84 | 0.84 | 0.84 | 0.84 | 0.84 | 0.84 | 0.84 | 0.84 | 1.68 | 2.52 | 4.20 | 4.20 |
| H 100mg dt | 8.13 | 4.65 | 1.27 | 2.54 | 3.80 | 5.07 | 5.07 | 7.61 | 7.61 | 10.14 | 10.14 | 11.41 | 15.21 |
| Z 400mg tab | 1.30 | 0.59 | 0.29 | 0.29 | 0.58 | 0.58 | 0.58 | 1.17 | 1.17 | 1.46 | 1.75 | 2.33 | 2.33 |

Pricing source: Global Drug Facility October 2023. Costs represented reflect mean average within stated age or weight category. Daily dosing recommendations: WHO operational handbook on tuberculosis. Module 5: management of tuberculosis in children and adolescents, 2022.WHO: World Health Organization; GDF: Global Drug Facility; US$: United States Dollar (not adjusted for purchasing power parity); MDR-TB: Multi-drug resistant tuberculosis

BDQ-bedaquiline, CFZ-clofazimine, CS-cycloserine, DLM-delamanid, E-ethambutol, ETO-ethionamide, hH-high dose isoniazid, LFX-levofloxacin, LZD-linezolid, MFX-moxifloxacin, PA-pretomanid, PAS-para-aminosalicylic acid, PTO-prothionamide, Pyr(B6)-Pyridoxine, TRD-terizidone, Z-pyrazinamide. Tab: tablet; cap: capsule; dt: dispersible tablet

**Formulations costed but not included in summary regimen analysis as alternative formulation used*

# APPENDIX 3: [TABLE] COST PER MONTH OF RR-TB PAEDIATRIC REGIMENS BY SETTING, FORMULATION AND AGE

| **Regimen** | **Setting*** | **Treatment duration** | **Cost (US$) per month by age group** | | | |
| --- | --- | --- | --- | --- | --- | --- |
|  |  |  | **Adult formulations** | | **Child-friendly formulations** | |
|  |  |  | **<15 years** | **<5 years** | **<15 years** | **<5 years** |
| **SHORT BEDAQUILINE-CONTAINING ALL ORAL REGIMENS** | | | | | | |
| BDQ (6)-LFX-CFZ-Z-E-hH-ETO (4)/ LFX-CFZ-Z-E (5) | Global (WHO) India | 9 | 27 | 17 | 89 | 44 |
| LZD (2)-BDQ (6)-LFX-CFZ-Z-E-hH (4)/ LFX-CFZ-Z-E (5) | Global (WHO) South Africa | 9 | 26 | 16 | 87 | 42 |
| BDQ-LFX-CFZ-Z-E-hH-ETO (6)/ LFX-CFZ-Z-E (5) | Global (WHO) India | 11 | 26 | 16 | 86 | 42 |
| LZD (2)-BDQ-LFX-CFZ-Z-E-hH (6)/ LFX-CFZ-Z-E (5) | Global (WHO) South Africa | 11 | 24 | 15 | 82 | 40 |
| *Mid-point* | | | 26 | 16 | 86 | 42 |
| **LONGER ALL ORAL REGIMENS** | | | | | | |
| **Fluoroquinolone susceptible – non severe** | | | | | | |
| LZD (2)-BDQ-LFX-CFZ (15) | Global (WHO) | 15 | 25 | 17 | 63 | 31 |
| BDQ-LFX-CFZ-CS (9) | Global (Sentinel Project) | 9 | 38 | 24 | 103 | 53 |
| LZD (2)-BDQ (6)-LFX-CFZ-TRD (15) | South Africa | 15 | 104 | 64 | - | - |
| LZD (2)-LFX-CFZ-TRD-PAS (15) | South Africa | 15 | 161 | 100 | - | - |
| BDQ-LZD (6)-LFX-CFZ-CS (15) | India | 15 | 32 | 20 | - | - |
| LZD-Z (6)-LFX-CFZ-CS (15) | India | 15 | 27 | 16 | - | - |
| LZD (6)-BDQ-LFX-CFZ-CS (12) | Philippines | 12 | 39 | 25 | - | - |
| LZD (6)-LFX-CFZ-CS-PAS (12) | Philippines | 12 | 90 | 56 | - | - |
| *Mid-point* | | | 65 | 40 | 83 | 42 |
| **Fluoroquinolone susceptible – severe** | | | | | | |
| LZD (2)-BDQ-LFX-CFZ-CS (20) | Global (WHO) | 20 | 37 | 24 | 101 | 52 |
| LZD (2)-BDQ-LFX-CFZ-CS (12) | Global (Sentinel Project) | 12 | 38 | 24 | 104 | 53 |
| LZD (2)-BDQ-LFX-CFZ-TRD (18) | South Africa | 18 | 110 | 69 | - | - |
| LZD (2)-LFX-CFZ-TRD-PAS (18) | South Africa | 18 | 161 | 100 | - | - |
| LZD (6)-BDQ-LFX-CFZ-CS (18) | India | 18 | 38 | 25 | - | - |
| LZD (6)-LFX-CFZ-CS-Z (18) | India | 18 | 28 | 17 | - | - |
| LZD (6)-BDQ-LFX-CFZ-CS (18) | Philippines | 18 | 38 | 25 | - | - |
| LZD (6)-LFX-CFZ-CS-PAS (18) | Philippines | 18 | 89 | 56 | - | - |
| *Mid-point* | | | 67 | 42 | 102 | 53 |
| **Fluoroquinolone resistant – non severe** | | | | | | |
| LZD (2)-BDQ-CFZ-CS (15) | Global (WHO) | 15 | 36 | 23 | 88 | 46 |
| BDQ-CFZ-CS-DLM (9) | Global (Sentinel Project) | 9 | 139 | 119 | 244 | 146 |
| LZD (2)-DLM (6)-BDQ-CFZ-TRD (15) | South Africa | 15 | 150 | 107 | - | - |
| LZD (2)-CFZ-TRD-DLM-PAS (15) | South Africa | 15 | 262 | 195 | - | - |
| LZD (6)-BDQ-CFZ-CS-DLM (12) | Philippines | 12 | 140 | 120 | - | - |
| LZD (6)-CFZ-CS-DLM-PAS (12) | Philippines | 12 | 191 | 151 | - | - |
| *Mid-point* | | | 153 | 119 | 166 | 96 |
| **Fluoroquinolone resistant – severe** | | | | | | |
| LZD (2)-BDQ-CFZ-CS-DLM (20) | Global (WHO) | 20 | 138 | 119 | 242 | 145 |
| LZD (2)-BDQ-CFZ-CS-DLM (12) | Global (Sentinel Project) | 12 | 139 | 119 | 245 | 146 |
| LZD (2)-BDQ-CFZ-TRD-DLM (18) | South Africa | 18 | 211 | 164 | - | - |
| LZD (2)-CFZ-TRD-DLM-PAS (18) | South Africa | 18 | 262 | 195 | - | - |
| LZD (6)-BDQ-CFZ-CS-DLM (18) | Philippines | 18 | 139 | 120 | - | - |
| LZD (6)-CFZ-CS-DLM-PAS (18) | Philippines | 18 | 190 | 151 | - | - |
| *Mid-point* | | | 180 | 145 | 243 | 145 |
| **Potential future regimens** | | | | | | |
| LZD (2) - BDQ-DLM-LFX (6) | Global (potential future regimen) | 6 | 130 | 117 | 249 | 142 |
| LZD (2) - BDQ-DLM-CFZ (6) |  | 6 | 141 | 123 | 248 | 144 |
| BDQ-PA-LZD (6) |  | 6 | 65 | 58 | 125 | 79 |
| BDQ-PA-LZD-MFX (6) |  | 6 | 70 | 63 | 142 | 89 |
| *Mid-point* | | | 102 | 90 | 191 | 114 |

Pricing source: Global Drug Facility October 2023;

Costs reflect mean costs per regimen adjusted for distribution of weight-for age in each category.

*Setting indicates the source of the guidance – either from National Programs (South Africa, Philippines or India) or Global recommendations (WHO or Sentinel), acknowledging that in some instances, local regimen choice may draw on national or global guidance.

BDQ-bedaquiline, CFZ-clofazimine, CS-cycloserine, DLM-delamanid, E-ethambutol, ETO-ethionamide, hH-high dose isoniazid, LFX-levofloxacin, LZD-linezolid, MFX-moxifloxacin, PAS-para-aminosalicylic acid, PTO-prothionamide, Pyr(B6)-Pyridoxine, TRD-terizidone, Z-pyrazinamide

WHO: World Health Organization; GDF: Global Drug Facility; US$: United States Dollar (not adjusted for purchasing power parity); RR-TB: Rifampicin resistant tuberculosis

# APPENDIX 4: [TABLE] TOTAL COST BY NATIONAL GUIDANCE FOR RR-TB PAEDIATRIC REGIMENS, PPP-ADJUSTED

| **Setting (local procurement arrangement)** | **Regimen** | **Treatment duration** | **Total regimen cost ($I) by age group** | |
| --- | --- | --- | --- | --- |
|  |  |  | **Adult formulations** | |
|  |  |  | **<15 years** | **<5 years** |
| **SHORT BEDAQUILINE-CONTAINING ALL ORAL REGIMENS** | |  |  |  |
| India (GDF) | BDQ (6)-LFX-CFZ-Z-E-hH-ETO (4)/ LFX-CFZ-Z-E (5) | 9 | 834 | 517 |
| South Africa (MHPL) | LZD (2)-BDQ (6)-LFX-CFZ-Z-E-hH (4)/ LFX-CFZ-Z-E (5) | 9 | 819 | 509 |
| India (GDF) | BDQ-LFX-CFZ-Z-E-hH-ETO (6)/ LFX-CFZ-Z-E (5) | 11 | 980 | 600 |
| South Africa (MHPL) | LZD (2)-BDQ-LFX-CFZ-Z-E-hH (6)/ LFX-CFZ-Z-E (5) | 11 | 930 | 568 |
| **LONGER ALL ORAL REGIMENS** | |  |  |  |
| **Fluoroquinolone susceptible – non severe** | |  |  |  |
| South Africa (MHPL) | LZD (2)-BDQ (6)-LFX-CFZ-TRD (15) | 15 | 2,619 | 1,612 |
| South Africa (MHPL) | LZD (2)-LFX-CFZ-TRD-PAS (15) | 15 | 6,141 | 3,809 |
| India (GDF) | BDQ-LZD (6)-LFX-CFZ-CS (15) | 15 | 1,662 | 1,038 |
| India (GDF) | LZD-Z (6)-LFX-CFZ-CS (15) | 15 | 1,412 | 848 |
| Philippines (GDF) | LZD (6)-BDQ-LFX-CFZ-CS (12) | 12 | 1,360 | 883 |
| Philippines (GDF) | LZD (6)-LFX-CFZ-CS-PAS (12) | 12 | 3,119 | 1,948 |
| **Fluoroquinolone susceptible – severe** | |  |  |  |
| South Africa (MHPL) | LZD (2)-BDQ-LFX-CFZ-TRD (18) | 18 | 3,527 | 2,229 |
| South Africa (MHPL) | LZD (2)-LFX-CFZ-TRD-PAS (18) | 18 | 7,360 | 4,563 |
| India (GDF) | LZD (6)-BDQ-LFX-CFZ-CS (18) | 18 | 2,346 | 1,515 |
| India (GDF) | LZD (6)-LFX-CFZ-CS-Z (18) | 18 | 1,718 | 1,025 |
| Philippines (GDF) | LZD (6)-BDQ-LFX-CFZ-CS (18) | 18 | 1,984 | 1,282 |
| Philippines (GDF) | LZD (6)-LFX-CFZ-CS-PAS (18) | 18 | 4,649 | 2,896 |
| **Fluoroquinolone resistant – non severe** | |  |  |  |
| South Africa (MHPL) | LZD (2)-DLM (6)-BDQ-CFZ-TRD (15) | 15 | 4,380 | 3,311 |
| South Africa (MHPL) | LZD (2)-CFZ-TRD-DLM-PAS (15) | 15 | 10,049 | 7,573 |
| Philippines (GDF) | LZD (6)-BDQ-CFZ-CS-DLM (12) | 12 | 4,868 | 4,187 |
| Philippines (GDF) | LZD (6)-CFZ-CS-DLM-PAS (12) | 12 | 6,627 | 5,252 |
| **Fluoroquinolone resistant – severe** | |  |  |  |
| South Africa (MHPL) | LZD (2)-BDQ-CFZ-TRD-DLM (18) | 18 | 8,217 | 6,747 |
| South Africa (MHPL) | LZD (2)-CFZ-TRD-DLM-PAS (18) | 18 | 12,050 | 9,080 |
| Philippines (GDF) | LZD (6)-BDQ-CFZ-CS-DLM (18) | 18 | 7,246 | 6,239 |
| Philippines (GDF) | LZD (6)-CFZ-CS-DLM-PAS (18) | 18 | 9,911 | 7,853 |

Costs represented reflect mean average, adjusted to reflect distribution of weight-for age in each category.

BDQ-bedaquiline, CFZ-clofazimine, CS-cycloserine, DLM-delamanid, E-ethambutol, ETO-ethionamide, hH-high dose isoniazid, LFX-levofloxacin, LZD-linezolid, MFX-moxifloxacin, PA-pretomanid, PAS-para-aminosalicylic acid, PTO-prothionamide, Pyr(B6)-Pyridoxine, TRD-terizidone, Z-pyrazinamide

GDF-Global Drug Facility (October 2023), MHPL – Master Health Product List (South African National Tender, February 2024)); WHO – World Health Organization; RR-TB – Rifampicin-Resistant Tuberculosis; $I: International dollar – a unit of currency that would buy a comparable amount of goods or services that a US dollar would buy in the United States; PPP – Purchasing power parity

# APPENDIX 5: [TABLE] TOTAL COST OF WHO-RECOMMENDED RR-TB PAEDIATRIC REGIMEN IN SOUTH AFRICA, INDIA AND PHILIPPINES (PPP-ADJUSTED)

| **Regimen** | **Age group** | **Total regimen cost by setting ($I** **)** | | |
| --- | --- | --- | --- | --- |
|  |  | **South Africa** | **India** | **Philippines** |
| **SHORT BEDAQUILINE-CONTAINING ALL ORAL REGIMEN** | | | | |
| BDQ (6)-LFX-CFZ-Z-E-hH-ETO (4)/ LFX-CFZ-Z-E (5) | | | | |
| Child formulation | <15 years | 1,885 | 2,755 | 2,326 |
|  | <5 years | 924 | 1,351 | 1,140 |
| Adult formulation | <15 years | 571 | 834 | 704 |
|  | <5 years | 353 | 517 | 436 |
| **LONGER ALL ORAL REGIMENS** | | | | |
| **Fluoroquinolone susceptible – non severe: LZD (2)-BDQ-LFX-CFZ (15)** | | | | |
| Child formulation | <15 years | 2,228 | 3,256 | 2,749 |
|  | <5 years | 1,081 | 1,581 | 1,335 |
| Adult formulation | <15 years | 892 | 1,304 | 1,101 |
|  | <5 years | 584 | 854 | 721 |
| **Fluoroquinolone susceptible – severe: LZD (2)-BDQ-LFX-CFZ-CS (20)** | | | | |
| Child formulation | <15 years | 4,738 | 6,926 | 5,847 |
|  | <5 years | 2,441 | 3,568 | 3,012 |
| Adult formulation | <15 years | 1,743 | 2,548 | 2,151 |
|  | <5 years | 1,119 | 1,636 | 1,381 |
| **Fluoroquinolone resistant – non severe: LZD (2)-BDQ-CFZ-CS (15)** | | | | |
| Child formulation | <15 years | 3,082 | 4,506 | 3,804 |
|  | <5 years | 1,635 | 2,390 | 2,018 |
| Adult formulation | <15 years | 1,270 | 1,857 | 1,568 |
|  | <5 years | 825 | 1,206 | 1,018 |
| **Fluoroquinolone resistant – severe: LZD (2)-BDQ-CFZ-CS-DLM (20)** | | | | |
| Child formulation | <15 years | 11,353 | 16,597 | 14,011 |
|  | <5 years | 6,796 | 9,935 | 8,386 |
| Adult formulation | <15 years | 6,480 | 9,474 | 7,997 |
|  | <5 years | 5,582 | 8,160 | 6,888 |

Costs represented reflect mean average, adjusted to reflect distribution of weight-for age in each category.

Pricing source: Global Drug Facility (GDF) October 2023;

BDQ-bedaquiline, CFZ-clofazimine, CS-cycloserine, DLM-delamanid, E-ethambutol, ETO-ethionamide, hH-high dose isoniazid, LFX-levofloxacin, LZD-linezolid, MFX-moxifloxacin, PAS-para-aminosalicylic acid, PTO-prothionamide, Pyr(B6)-Pyridoxine, TRD-terizidone, Z-pyrazinamide

$I: International dollar – a unit of currency that would buy a comparable amount of goods or services in a specific country that a US dollar would buy in the United States

WHO: World Health Organization; GDF: Global Drug Facility; US$: United States Dollar; RR-TB: rifampicin- resistant tuberculosis

# APPENDIX 6: [FIGURE] PROPORTIONAL CONTRIBUTION OF INDIVIDUAL CHILD-FRIENDLY AND ADULT FORMULATIONS TO TOTAL PAEDIATRIC RR-TB REGIMEN COST

Pricing source: Global Drug Facility (GDF) October 2023;

BDQ-bedaquiline, CFZ-clofazimine, CS-cycloserine, DLM-delamanid, E-ethambutol, ETO-ethionamide, hH-high dose isoniazid, LFX-levofloxacin, LZD-linezolid, MFX-moxifloxacin, , PAS-para-aminosalicylic acid, PTO-prothionamide, Pyr(B6)-Pyridoxine, TRD-terizidone, Z-pyrazinamide

AF: Adult Formulation; CF: Child Formulation. m: length of regimen in months. WHO: World Health Organization-recommended regimen

# APPENDIX 7: [FIGURE] WHO RR-TB REGIMEN COST AS A FACTOR OF DOMESTIC GENERAL GOVERNMENT HEALTH EXPENDITURE, PER CAPITA (GDF PROCUREMENT, PPP ADJUSTED in <15 YEARS AGE GROUP)

BDQ-bedaquiline, CFZ-clofazimine, CS-cycloserine, DLM-delamanid, E-ethambutol, ETO-ethionamide, hH-high dose isoniazid, LFX-levofloxacin, LZD-linezolid, MFX-moxifloxacin, PA-pretomanid, PAS-para-aminosalicylic acid, PTO-prothionamide, Pyr(B6)-Pyridoxine, TRD-terizidone, Z-pyrazinamide

GDF-Global Drug Facility, US$-United States Dollar; WHO – World Health Organization; RR-TB – Rifampicin-Resistant Tuberculosis; PPP – Purchasing Power Parity

# APPENDIX 8: SOURCE GUIDANCE FOR INCLUDED REGIMEN:

**World Health Organization (WHO)** (1)

The WHO recommendations for the treatment of RRR-TB in children was updated in 2022. It recommends prioritising the short, standardised all-oral bedaquiline-containing regimen for all children and adolescents with RR-TB, and using longer, individualised regimens for children and adolescents not eligible for the short regimen (1). Due to the lack of safety data on pretomanid in children aged below 14 years, the BPaLM/BPaL regimen (a combination of bedaquiline, pretomanid, linezolid and moxifloxacin), BPaL (bedaquiline, pretomanid and linezolid) is only recommended for adults and adolescents aged 14 years and older (2). This regimen has been included in this analysis for reference to a potential future scenario where it will be a routine recommendation for children. The 2022 WHO guideline suggests that the duration of treatment with individualised regimens in children should be determined based on the site and severity of disease and the extent of resistance, and that children with non-severe disease can usually be treated for less than 18 months while children with extensive disease may require longer treatment durations (1).

For the analysis, the duration of the short regimens was assumed as 9-11 months (depending on the timing of smear conversion) and for the longer regimens an average treatment duration of 15 months was assumed for non-severe cases and 20 months for severe cases. It was also assumed that linezolid (if included in a regimen) would be provided for two months regardless of the overall length of the chosen regimen due to the increase in adverse events after two months. Pyridoxine (vitamin B6) supplementation was assumed if high-dose isoniazid was included in a regimen.

The Sentinel Project on Paediatric Drug-Resistant Tuberculosis (3)

The Sentinel Project on Pediatric Drug-Resistant Tuberculosis is a global partnership of researchers, caregivers, and advocates who share evidence and resources that can increase children’s access to prompt and effective treatment (<http://sentinel-project.org/about/>). The fifth edition of the Management of Multidrug-Resistant Tuberculosis in Children: A Field Guide (3) was released in 2022. The guide offers practical recommendations on the diagnosis, treatment, and monitoring of children with drug-resistant tuberculosis based on the best available research evidence and clinical experience.

The Field Guide offers a similar approach to the individual regimen composition recommended by the WHO but suggest shorter treatment durations for both severe and non-severe disease: 6-9 months and 9-12 months, respectively. In addition, the field guide suggests that the inclusion and duration of use of linezolid can be determined based on disease severity and risk factors for toxicity, with a duration of eight weeks proposed for patients with severe, fluoroquinolone-susceptible RR-TB.

For the analysis, a treatment duration of nine months was assumed for non-severe disease and twelve months for severe disease, and linezolid was included in regimens for the treatment of severe RR-TB disease for a duration of eight weeks.

**South Africa**

Multiple South African national guidelines suggest approaches to managing patients with drug-resistant TB (4–8) of which the ‘Management of rifampicin-resistant tuberculosis: A clinical reference guide’(6) was published the most recently in 2019. The guide was developed when there was limited evidence on the use of bedaquiline and delamanid in children, so these two medicines were not included in many regimens for children aged <6 years. Use of the short, standardised WHO regimen that includes linezolid is recommended for children aged >6 years. For children aged six years and above who do not meet the short regimen inclusion criteria, and children aged <6 years, treatment with a longer, individualised regimen is recommended, which may consist of four to five drugs given for a period of six months, followed by three or four medicines (for non-severe or severe disease, respectively) for the rest of the treatment period. The reference guide (6) propose that individualised regimen construction should be based on the WHO approach and prioritise the WHO Group A and B drugs. It is recommended that children aged <6 years will receive a regimen consisting of at least four effective drugs and include delamanid in children aged 3 years and above. The guide suggests a longer regimen treatment duration of 9-15 months for non-severe and 12-18 months for severe disease in patients aged <6 years, and a treatment duration of eighteen months for all children aged >6 years. For completeness, the analysis detailed the costs of recommended regimen across all ages under 15; this provides some costs for ages where a medicine may not be recommended in a particular context, but will inform costs if age restrictions in national guidance are amended in future.

For the analysis, a treatment duration of 15 months was assumed for non-severe disease and 18 months for severe disease, irrespective of age. As with the WHO and Sentinel Project guidance, it was assumed that linezolid (if included in a regimen) would be provided for two months regardless of the overall length of the chosen regimen. Pyridoxine (vitamin B6) supplementation was assumed if high-dose isoniazid or terizidone was included in a regimen, as recommended in the guide. When constructing a longer regimen sample for children aged <6 years with fluoroquinolone-susceptible disease, drugs recommended for inclusion in both the 0-3 year and 3–6 year groups were selected by the analyst.

**India**

The Paediatric TB Management Guidelines 2022 (9) reflects the most up to date national guidance on the management of paediatric TB (including RR-TB) in India. Use of bedaquiline in children <5 years and delamanid in children aged <6 years is not yet recommended in this guideline, and delamanid may only considered as a replacement in longer oral regimens.

Four standard regimens for fluoroquinolone-susceptible RR-TB are proposed in the guideline based on age and severity of disease. Children aged over five years (and weighing over 15kg) with non-severe pulmonary RR-TB are eligible for the short all-oral bedaquiline-containing WHO regimen that includes ethionamide, while children with disseminated/ extensive/ severe pulmonary disease will receive a standardised, longer all-oral regimen which can be modified with expert input. For children aged <5 years, a short regimen that includes an injectable (kanamycin or amikacin) is recommended for non-severe pulmonary RR-TB^[[1]](#footnote-1)^ and a modified^[[2]](#footnote-2)^ longer all-oral regimen is recommended when there is disseminated/ extensive/ severe pulmonary disease. The longer RR-TB regimen is constructed using the WHO approach (prioritising the WHO Group A and B drugs) and should include at least 4 to 5 effective medicines to which the Mycobacterium tuberculosis strain is known or likely to be susceptible in the initial 6 to 8 months, and at least 3-4 drugs in the last 12 months. The guide suggests a treatment duration of 18-20 months for children with severe pulmonary disease, but shortening the duration to 12-18 months can be considered depending on treatment response.

For the analysis, a treatment duration of 15 months was assumed for non-severe disease and 18 months for severe disease. It was assumed that linezolid is only used in the first six months (time point when dose tapering is considered according to guideline) due to neurologic adverse events. For both non-severe and severe disease, use of 5 medicines in first 6 months is assumed, followed by use of 4 and 3 medicines for rest of the treatment duration for severe and non-severe disease respectively. Pyridoxine (vitamin B6) supplementation was assumed if high-dose isoniazid was included in a regimen.

**The Philippines**

The updated National Tuberculosis Control Program: Manual of procedures for frontline health workers (6^th^ edition) was published in 2020 (10). The guideline recommends that a regimen consisting of four to five drugs is given for the entire treatment duration in the treatment of children with RR-TB aged under 15 years. Standardised treatment regimens, constructed based on fluoroquinolone-resistance and age, are proposed in the manual, with severity of disease determining the treatment duration (9-12 months for non-severe disease, depending on clinical progress, and 15-18 months for severe or extensive disease). The use of bedaquiline in children aged < 6 years and delamanid in children aged < 3 years require consultation with the TB Medical Advisory Committee, so its use is limited.

In the costing analysis the upper range of the treatment duration was used for non-severe disease (12 months) and severe disease (18 months). The drugs recommended for inclusion in both the 0-3 year and 3–6 year-old groups were selected for children in the combined <6 age bracket. It was assumed that linezolid is only used in the first six months, and all patients taking linezolid received pyridoxine daily as per the manual (although it is recognised that concomitant pyridoxine may not be practiced in all settings).

**Potential future regimen**

Globally there is a move towards shorter treatment regimens for children to align with practice in adults and in treating drug-susceptible TB in children, particularly related to six-month BPaL (bedaquiline, pretomanid, linezolid) and BPaLM (bedaquiline, pretomanid, linezolid, moxifloxacin) regimens.. In order to reflect possible future treatment scenarios and new evidence relating to safety and efficacy in children emerges, this analysis has also included the following regimen:

LZD (2) - BDQ-DLM-LFX (6)

LZD (2) - BDQ-DLM-CFZ (6)

BDQ-PA-LZD (6) – (BPaL regimen)

BDQ-PA-LZD-MFX (6) – (BpaLM regimen)

In the above regimen the standard bedaquiline loading dose is used followed by a once daily dosing throughout the maintenance period

# APPENDIX 9: [TABLE] PROPORTION OF POPULATION FALLING IN WEIGHT BANDS USED IN THE ANALYSIS:

| **Weight range (kg)** | **Corresponding months of life*** | **Proportion of population aged <15 years** | **Proportion of population aged <5 years** |
| --- | --- | --- | --- |
| **3 to <5** | m0-m2 | 1.16% | 3.45% |
| **5 to <7** | m3-m6 | 2.33% | 6.89% |
| **7 to <10** | m7-m19 | 7.42% | 21.96% |
| **10 to <12** | m20-m30 | 6.16% | 18.23% |
| **12 to <16** | m31-m56 | 14.49% | 42.89% |
| **16 to <20** | m57-m80 | 13.30% | 6.58% |
| **20 to <24** | m81-m101 | 11.61% |  |
| **24 to <30** | m102-m125 | 13.25% |  |
| **30 to <36** | m126-m144 | 10.47% |  |
| **36 to <46** | m145-m172 | 15.41% |  |
| **46 and over** | m173-m180 | 4.40% |  |
| **All children <15 years** | m0-m180 | 100.00% |  |
| **All children <5 years** | m0-m60 | 33.78% | 100.00% |

### *Applying World Health Organization child growth standards (11,12) under an assumption that children receiving treatment for tuberculosis will have a weight for age one standard deviation below average weight for age in the general childhood population.

References

1. World Health Organization. WHO operational handbook on tuberculosis. Module 5: management of tuberculosis in children and adolescents. Geneva; 2022.

2. World Health Organization. WHO consolidated guidelines on tuberculosis. Module 4: Treatment - drug-resistant tuberculosis treatment, 2022 update. Geneva; 2022.

3. The Sentinel Project for Pediatric Drug-Resistant Tuberculosis. Management of Drug-Resistant Tuberculosis in Children: A Field Guide (fifth edition) [Internet]. Boston, USA; 2022 Nov [cited 2023 May 2]. Available from: http://sentinel-project.org/wp-content/uploads/2022/04/DRTB-Field-Guide-2021_v5.1.pdf

4. National Department of health. Standard Treatment Guidelines and Essential Medicines List for South Africa: Hospital level paediatrics. 2017 Edition. Pretoria: Department of Health; 2017.

5. National Department of Health. Guidelines for the management of tuberculosis in children 2013. Pretoria; 2013.

6. South African Department of Health. Management of rifampicin-resistant tuberculosis: A clinical reference guide. Pretoria; 2019.

7. Department of Health. Management of drug-resistant tuberculosis - Policy guidelines (updated 2013). Pretoria; 2012.

8. Department of Health. National Tuberculosis Management Guidelines 2014 [Internet]. Pretoria; 2014 [cited 2023 Mar 10]. Available from: https://www.knowledgehub.org.za/elibrary/national-tuberculosis-management-guidelines

9. National Tuberculosis Elimination Programme. Paediatric TB Management Guideline 2022 (draft). New Delhi; 2022.

10. National Tuberculosis Control Program. National Tuberculosis Control Program: Manual of procedures (6th edition). Manila; 2020.

11. World Health Organization. WHO growth reference data (children aged 5-19 years) [Internet]. Geneva: World Health Organization; 2007 [cited 2023 May 2]. Available from: https://www.who.int/tools/growth-reference-data-for-5to19-years

12. World Health Organization. WHO child growth standards (children aged 0-5 years) [Internet]. Geneva: World Health Organization; 2006 [cited 2023 May 2]. Available from: https://www.who.int/tools/child-growth-standards

1. Regimen not included in the study analysis as not an all-oral regimen [↑](#footnote-ref-1)
2. Modification to longer all-oral regimen for children aged >5 years: regimen should not include bedaquiline, so replacement of bedaquiline in the sequence of amikacin, pyrazinamide, ethionamide, para-aminosalicylic acid, ethambutol, penems is recommended [↑](#footnote-ref-2)
