# Supplementary material for: Pharmaceutical cost dynamics for the treatment of rifampicin-resistant tuberculosis in children and adolescents in South Africa, India, and the Philippines
Source: PLoS One. 2024 Jul 23;19(7):e0305930. doi: 10.1371/journal.pone.0305930 (PMC11265651; doi:10.1371/journal.pone.0305930)
Supplement: Attachment — Submitted filename: Response to reviewers 20240516.docx [file pone.0305930.s002.docx]

Reviewer comments CATALYST paper:

Thank you for the comprehensive and detailed comments, all well received. We have no major rebuttal points to make and have described in the table below how we have addressed each comment. Where the reviewer comment is very straightforward and specific we have simply noted “change made” to indicate that the suggested change has been made as directed.

| No. | **Reviewer 1 comment** | **Author’s response** |
| --- | --- | --- |
| 1.1 | Update references (and corresponding information and estimates), when appropriate (eg, Global TB report, latest GDF catalogue) | Agree, changes made. |
| 1.2 | Carry out a detailed language check throughout | Agree, changes made throughout manuscript. |
| 1.3 | In the introduction (or the discussion?), I would recommend adding a couple of sentences on the concept of cost-based generic prices and add a reference to Hill A, Barber M, Gotham D. Estimated costs of production and potential prices for the WHO Essential Medicines List. BMJ Global Health | Agree, added the cost-plus pricing concept, and cited the recommended reference – we consider that this fits best in the discussion in section related to factors that may reduce prices. |
| 1.4 | In the discussion, I recommend adding a couple of statements on the risk of using adult formulations (even if they may end up being cheaper for programmes) when they are manipulated for administration in children (under/over dosing etc). | Agree, added text to discussion. |
| 1.5 | Include considerations around “future regimens” (but I encourage you to change this terminology) in the discussion, as this part is totally missing now. Include some forward-looking considerations on the affordability of such regimens if recommended by WHO in the context of the other regimens you describe. | Agree, added further considerations particularly on affordability in discussion, and adjusted terminology to refer to the regimen consistently as “adapted BPaL/BPaLM regimen”, noting that it is described that these are not currently recommended regimen |
| 1.6 | ***ABSTRACT***  “Providing child-friendly formulations of medicines in the <5-year-old age group across all WHO recommended regimen is expected to cost an additional $380 (range $212-$563) per child” – please, add some short text on the added value of investing in this despite the additional cost | Agree added |
|  | ***INTRODUCTION*** |  |
| 1.7.1 | Update reference 1 and estimated incident cases with data from the latest Global TB report 2023 (including for Table 1) | Agree, change made. |
| 1.7.2 | Update reference 8 with the latest GDF catalogue available and update link too. | Agree, change made. |
| 1.7.3 | Page 12, add a relevant reference to the CATALYST trial and very briefly provide an update on its status to better contextualize this specific study. | Agree, done, noting the CATALYST trial is completed. |
| 1.7.4 | Page 13, the sentence “Purchasing through GDF can result in reduced prices and improved continuity of supply than could be achieved through individual country procurement” – what is it meant with “continuity of supply than could be achieved through individual country procurement”? | Agree text can be clearer. Sentence edited. The intent of the sentence is to note that the GDF procurement mechanism may provide more certainty for the supply-chain as larger volumes are purchased, as GDF manages supply (and can address supply problems) on an international (rather than local) basis. |
| 1.7.5 | Page 13, second paragraph, rephrase with “The GDF catalogue includes formulations of TB medicines for adults and children, with pricing and formulation information regularly updated” | Agree and thank you. Updated as suggested. |
| 1.7.6 | Page 13, second paragraph – please provide updated estimates of countries procuring through national programmes, if possible. The current information you provide is from 2021, which is relatively old. Also, perhaps it would be good to add a couple of statements on the nuances between procuring first-line medicines (previously supported by donors and done via GDF by most countries, but now mostly procured with local budget and not anymore from GDF) and second-line medicines. | Agree and thank you. Updated as suggested with no. of countries using GDF in 2023. |
| 1.7.7 | Page 13, second paragraph – replace “only some are accessing child-friendly formulations” with “are procuring child-friendly formulations” | Agree – removed this sentence when editing to address comment above. |
|  | ***METHODS*** |  |
| 1.8.1 | Page 14, first para – what do you mean with “ingredients-based pharmaceutical cost analysis”? Perhaps it becomes clearer later on in the paper, but at this stage it is not. It sounds as if you have done an estimate of how much a certain formulation should cost based on the cost of the API, manufacturing + some revenue for the developer (but I do not think this is the case). | Thank you. “Ingredients-based costing” is an established term in costing methods relating to identifying the individual components of an intervention or service and aggregating to a summary cost. However, we acknowledge that given the focus of this paper there is possibility for confusion with pharmaceutical excipients. We consider that it is more straightforward to call this simply a “pharmaceutical cost analysis” as it is clear in our further description that this is an ingredients-based costing approach. |
| 1.8.2 | Page 14, first para – clarify what you mean with “from the perspective of national TB programmes in South Africa, India and the Philippines and at a global perspective”? | Thank you. We used the term “global perspective” to represent the instances where we present the cost (eg in US$) unadjusted for purchasing power parity (PPP). However, we consider it is best to remove the term “global perspective” as we are clear later in the paper about when we do and do not adjust for local county PPP. |
| 1.8.3 | Page 14, second para – propose rephrasing as “The RR-TB treatment regimens utilized in this cost-analysis included regimens as per global (…) and national (…) guidance, and potential future regimens based on authors’ judgement” | Thank you, agree and edited sentence with minor edit: “The RR-TB treatment regimens included in this analysis reflected global (…) and national (…)….” |
| 1.8.4 | Please, check the spelling of the methods section in general, eg, “severe regimens” does not make too much sense; “The BPaLM/BPaL (please, invert the order) adaption regimens (please, rephrase). | Agree, general check done, and specific adjustments made – we now refer to specific regimen in the treatment of “severe disease” or “non-severe disease” (rather than calling a regimen a “severe regimen”) which we agree makes much more sense. |
| 1.8.5 | Page 15 – what does it mean that “The BPaL/BPaLM adaption regimens are not currently recommended by WHO or any national TB treatment guidance for children “but reflect the opinions of a wide group of experienced child TB physicians” (please, rephrase, ie “are provided for comparison as potential future treatment scenarios, in line with experienced child TB physicians’ opinion on potential treatment regimens available for children in the future..) | Agree this suggestion makes it more clear change made. |
|  | ***DATA ANALYSIS*** |  |
| 1.9.1 | Page 16 – “Therefore, GDF pricing was used as indicative in the Indian context, under an assumption that prices are expected to be comparable between the Central TB Division and the GDF procurement mechanism” – what information you have to assume that prices are expected to be comparable between the Central TB Division and GDF? Is this assumption based on specific criteria, for example the high medicine volume procured by both India and GDF which could lead to the same prices? Please, clarify. | Agree and change made to add our criteria of high volume of medicine purchase and extensive domestic manufacturing in India. |
| 1.9.2 | Page 18 – “WHO formulation dosing recommendations were used to determine assumptions…” Please, rephrase. | Agree – simply terms this “WHO guidance” and cited, as it is implicit that the guidance is related to number of tablets per day etc. |
|  | ***RESULTS*** |  |
| 1.10.1 | Page 19 – what do you mean with “given limited specifications for these formulations in national guidance in South Africa, India, and the Philippines”? Does this relate to the lack of clarity on whether these paediatric formulations are available in the respective countries? Country guidelines usually do not include information on whether paediatric formulations of TB medicines are used or not. I strongly recommend looking into the national Essential Medicines Lists for these countries or retrieve this information elsewhere. | Agree and thank you. Yes, we were meaning the relevant national essential medicines lists/formularies and have made the correction. |
| 1.10.1 | Page 21 – “The standardised “short” regimens (9-11 months) are currently the preferred choice of treatment for RR-TB according to WHO as well as South African and Indian national TB guidelines for patients with uncomplicated, non-severe, fluoroquinolone-susceptible RR-TB disease”. According to WHO and if you do not specify the age, the preferred regimen is BPaLM/BPaL. Please, clarify this sentence possibly by specifying the age you refer to. | Agree and thank you for spotting this – we have corrected. We note that the exact age differs across guidance (eg South Africa differentiates for under 6s) so we have simply noted “children” rather than specify ages. However, in the supplementary appendix we have included detailed summary of the source guidance to justify the specification for each regimen used, including by age. |
|  | ***DISCUSSION*** |  |
| 1.11.1 | Page 29, second paragraph “particularly relating to the adoption of new treatment regimenS and new child friendly formulations.” | Agree, change made. |
| 1.11.2 | Page 29 – repetition of “this analysis” multiple times across these paragraphs – check language and rephrase. | Agree, revised language in these paragraphs. |
| 1.11.3 | Page 29, second paragraph “The analysis supports complements existing analysis in this area, notably the routine pricing analysis under the Médecins Sans Frontières Access Campaign “DR-TB Drugs Under the Microscope” that assesses the cost of adult and child TB medicines and regimens more generally (29)” | Agree, change made |
| 1.11.4 | Page 30, first paragraph “replace national treatment programs” with “national tuberculosis programmes” | Agree, change made |
| 1.11.5 | Page 30, “The child-friendly formulation price differential is also a direct consequence of procurement practices” – does this refer to what you specify in the following sentence, that is the it depends on the volumes procured? If this is the case, then “procurement practices” is not the best terminology to use. Please, rephrase with, eg, procurement volumes. | Agree, change made |
| 1.11.6 | Page 30 “it can be expected that economies of scale will lead to reduced prices, demonstrated by a 55% price reduction in the price of bedaquiline in August 2023 that applied only to the adult formulation (8)”. I would smoothen the language behind this statement. In general, it is true that as volumes increase, then you can negotiate better prices, but that’s not always the case. Plus, for bedaquiline, there were also strong advocacy pushes to lower the price (it is not just the increased volumes). | Agree, change made, also to incorporate the role of advocacy in reducing prices. |
| 1.11.7 | Page 32 – add a reference to back up this statement: “A consistent finding in formulation acceptability studies is that child friendly formulations are preferred by both children and caregivers”. | Agree, reference added |
|  |  |  |
|  | **Reviewer 2 comment** |  |
| 2.1 | While a straightforward concept, this article presents useful information on drug costing in children that may be useful to national TB programs to inform planning and budgeting. | Thank you. Yes agree. This article is in response to the identified need for this type of information for national TB programs |
| 2.2 | As mentioned in the discussion, the article could have been strengthened by a more comprehensive analysis on total costs e.g. costs to conduct basic minimum active drug safety monitoring (laboratory testing, ECG etc) and hospital costs (outpatient/hospitalisation etc). | Agree and as mentioned we have listed this in the discussion. We also note that the targeting of this research to pharmaceutical costing assists in the very specific analytical needs relating to decisions about which medicines and formulations to be made available. A more generalized costing that includes eg hospitalizations etc risks losing this detail. |
| 2.3 | The article is otherwise clearly written and presented. | Thank you. |
| 2.4 | As a minor point, references could also be updated e.g. 2023 global TB report instead of 2022 (ref 1). | Thank you, updated this and other refs in an overall update of the reference list*.* |
| 2.5 | Also page 14: it is not clear to me that costs were "modelled" ? were they not just calculated based on regimen and formulation costs. | Agree – replaced “modelled” with “calculated” |
|  |  |  |
|  | **Journal requirements/comment** |  |
| J1 | Please ensure adherence to PLOS One style requirements, including file naming, ref to template for [main body](https://journals.plos.org/plosone/s/file?id=wjVg/PLOSOne_formatting_sample_main_body.pdf) and [affiliations](https://journals.plos.org/plosone/s/file?id=ba62/PLOSOne_formatting_sample_title_authors_affiliations.pdf) | Changes made to:   - Affiliations - Names of files |
| J2 | Please note that funding information should not appear in any section or other areas of your manuscript. We will only publish funding information present in the Funding Statement section of the online submission form. Please remove any funding-related text from the manuscript. | Funding statement in body of manuscript removed. |
| J3 | When completing the data availability statement of the submission form, you indicated that you will make your data available on acceptance. We strongly recommend all authors decide on a data sharing plan before acceptance, as the process can be lengthy and hold up publication timelines. Please note that, though access restrictions are acceptable now, your entire data will need to be made freely accessible if your manuscript is accepted for publication. This policy applies to all data except where public deposition would breach compliance with the protocol approved by your research ethics board. If you are unable to adhere to our open data policy, please kindly revise your statement to explain your reasoning and we will seek the editor's input on an exemption. Please be assured that, once you have provided your new statement, the assessment of your exemption will not hold up the peer review process. | We note that as this is a costing analysis, we attempted to ensure that all applicable and relevant data is included in either the main text or supplementary appendices. This includes data down to the level of the individual unit price per medicine formulation. We note that a significant amount of data we have used is already in the public domain as referenced. We also note that both Reviewers noted that all the data underlying the findings have been made available.  However, we are more than happy to provide any additional information as required (such as back-end calculations) and will seek confirmation from you on the level and format of any additional data required. |
| J4 | Please include captions for your Supporting Information files at the end of your manuscript, and update any in-text citations to match accordingly. Please see our Supporting Information [guidelines](http://journals.plos.org/plosone/s/supporting-information) for more information | Added captions and updated in-text citations in line with guidance provided. |
| J5 | Please review your reference list to ensure that it is complete and correct. If you have cited papers that have been retracted, please include the rationale for doing so in the manuscript text, or remove these references and replace them with relevant current references. Any changes to the reference list should be mentioned in the rebuttal letter that accompanies your revised manuscript. If you need to cite a retracted article, indicate the article’s retracted status in the References list and also include a citation and full reference for the retraction notice | To our knowledge we have not cited any retracted papers. |
